# Open questions: microbes, metabolism and host-pathogen interactions

**DOI:** 10.1186/1741-7007-12-18

**Published:** 2014-03-28

**Authors:** Malcolm McConville

**Affiliations:** 1 Department of Biochemistry and Molecular Biology, Bio21 Molecular Science and Biotechnology Institute, 30 Flemington Road, Parkville, VIC 3010, Australia

## Microbial metabolism - back in fashion

Science and fashion share some common traits, including a propensity to rediscover old ideas/themes and re-examine them in a new light. Such is the case with cellular metabolism. After the renaissance in metabolic studies during the mid-20th century, when many University science and medical departments were full of research groups working on enzymes and metabolic pathways, interest in cellular metabolism receded - overtaken by the revolutions in genomics, cell biology, the rise of the ‘omics’ technologies and perhaps a perception that metabolism was primarily a housekeeping process and a bit boring. However, we are now experiencing an impressive revival of interest in cellular metabolism. This has been driven, in part, by an appreciation that the metabolic activities of all cells are intimately tailored to the specific needs and functions of that cell or tissue and that detailed information on cellular metabolism is critical for understanding cellular development and disease mechanisms. It is also clear that there are major gaps in our understanding of how cellular metabolism is regulated *in vivo*. At the same time there have been impressive advances in our ability to measure metabolic processes *in vivo*, and to integrate ‘omics’ data in order to understand the multiple levels of regulation of metabolism.

The revival in interest in metabolism has been particularly apparent to those of us who work on microbial pathogens. These organisms are in a constant war with their host over access to carbon sources and nutrients and, like other cells, need to tailor their metabolism to fulfill particular tasks, such as the synthesis of specialized virulence factors or the maintenance of their energy/redox state in inhospitable environments. This battle is likely to have played a key role in defining microbial growth strategies and the range of host niches that each pathogen can occupy. The renewed interest in microbial metabolism has been prompted by the imperative (and increased funding opportunities in some areas) to identify new drug targets and/or understand the mechanism of action of existing drugs, as well as the realization that, in most cases, direct information on nutrient levels and pathogen metabolism *in vivo* is missing. Moreover, despite some of these pathogens’ being amongst the most intensively studied organisms in biology, we still do not know what the majority of their genes do, although it is reasonable to suggest that many are directly or indirectly involved in regulating metabolism. On the other side of the ledger, we are only just beginning to explore how intracellular and extracellular pathogens manipulate the metabolism of their host in order to create a more hospitable metabolic niche. Some of the open questions that come to mind when considering the role of metabolism in host-pathogen interactions are as follows.

## What carbon sources do microbial pathogens use *in vivo*?

Most baseline studies on microbial metabolism are undertaken on cultured stages grown in standard (often nutrient-rich) medium. Not surprisingly, there is increasing evidence that the metabolism of the same microbe can be completely different in infected tissues, reflecting differences in nutrient availability and the need to activate specific pathways in the face of complex host responses. A detailed understanding of microbial pathways that are being switched on or off during infection, the identity of specific nutrients that are being utilized and the extent to which there is redundancy in these pathways is essential for understanding microbial pathogenesis. Recent developments in advanced analytical and metabolomics approaches, the availability of fluorescent metabolite sensor and *in situ* metabolite imaging technologies will provide some of the tools needed to obtain this information. Interestingly, studies on core metabolism of microbial pathogens *in situ* are also starting to highlight significant differences in the enzymology and regulation of these pathways compared with those in their hosts, suggesting that even microbial pathways that were previously thought to be too similar to host pathways to be targeted are now valid drug targets.

## How do pathogens adapt to different host environments during chronic infections?

Many bacterial, fungal and protozoan pathogens are associated with long-term chronic infections where the pathogen persists for many years or the life-time of the host. With few exceptions, we know very little about the physiological state of the pathogens, or even where they reside, during these chronic infections. It is often assumed that they enter a hibernating/quiescent state, although there is increasing evidence that a number of so-called latent pathogen stages exhibit appreciable metabolic activity. We will need to better define the growth rate, physiology and metabolism of latent microbial stages in order to understand pathogenesis mechanisms (including the potential role of slow growth and quiescence in combating host microbicidal responses), and develop drugs that target processes essential for survival during latency.

## How do they sense their nutrient environment?

Microbial pathogens must be adept at sensing nutrient levels as well as other stresses in their environment. Nutrient sensing and signaling pathways are starting to be defined in many prokaryotic pathogens. However, much less is known about how this occurs in fungal and protozoan pathogens. Most protists, for example, lack membrane receptors common in higher eukaryotes and exhibit greatly reduced transcriptional regulation. As an extreme example, *Leishmania* parasites lack transcription factors and gene-specific transcriptional regulation. However, they retain a full suite of eukaryotic kinases and phosphatases that normally lead to activation of transcription factors. These observations suggest that many of these pathogens may have evolved new signaling modules that are quite different from the well characterized signaling cascades in their host. How and which nutrient signals are sensed, the identity of intervening signaling modules, and the number and type of down-stream intracellular effectors remain one of the big ‘black boxes’ in microbial molecular biology. The delineation of these pathways is likely to open a rich source of new drug targets, as well as potentially reveal regulatory mechanisms that have been conserved in all eukaryotes, but have been overlooked in higher eukaryotes with more complex transcriptional controls.

## How does host metabolism influence host-pathogen dynamics?

We know that many, if not all, pathogens directly or indirectly affect the growth and physiology of surrounding host cells. However, we know very little about how changes in host cell metabolism, mediated by the pathogen itself, the host immune response or global host nutrition affect the outcome of infections. Pathogens may manipulate host metabolism in order to increase their access to essential nutrients/carbon sources, or to down-regulate pathways that are needed to sustain host microbicidal processes. Intriguingly, we know that the effector functions of many immune cells (such as cytokine secretion, antigen presentation, cytotoxic responses) are dependent upon changes in their carbon metabolism. Not surprisingly, there is increasing evidence that microbial pathogens have developed strategies for altering the nutrient environment in infected tissues in order to modulate the host immune response. One of the major challenges for the future, therefore, is to develop a genuine systems-wide approach for measuring the intertwined cellular metabolism of host and pathogen *in situ*.

